# The Cost of Sex: Quantifying Energetic Investment in Gamete Production by Males and Females

**DOI:** 10.1371/journal.pone.0016557

**Published:** 2011-01-24

**Authors:** April Hayward, James F. Gillooly

**Affiliations:** Department of Biology, University of Florida, Gainesville, Florida, United States of America; King Abdullah University of Science and Technology, Saudi Arabia

## Abstract

The relative energetic investment in reproduction between the sexes forms the basis of sexual selection and life history theories in evolutionary biology. It is often assumed that males invest considerably less in gametes than females, but quantifying the energetic cost of gamete production in both sexes has remained a difficult challenge. For a broad diversity of species (invertebrates, reptiles, amphibians, fishes, birds, and mammals), we compared the cost of gamete production between the sexes in terms of the investment in gonad tissue and the rate of gamete biomass production. Investment in gonad biomass was nearly proportional to body mass in both sexes, but gamete biomass production rate was approximately two to four orders of magnitude higher in females. In both males and females, gamete biomass production rate increased with organism mass as a power law, much like individual metabolic rate. This suggests that whole-organism energetics may act as a primary constraint on gamete production among species. Residual variation in sperm production rate was positively correlated with relative testes size. Together, these results suggest that understanding the heterogeneity in rates of gamete production among species requires joint consideration of the effects of gonad mass and metabolism.

## Introduction

Much of sexual selection and life history theory is based on assumptions about the relative investment of males and females in sexual reproduction. The predominance of female choice in sexual selection, and parental care by females, is typically attributed to the greater investment by females per offspring [1], [2]. In many species, eggs are much larger than sperm (i.e. anisogamy) and thus sperm are often considered relatively “cheap” to produce [2], [3], [4], [5]. However, quantifying the energetic cost of gamete production both within and between the sexes has remained a difficult challenge since it includes both the investment in gonads and the rate of production of gamete biomass [4], [6], [7], [8]. A better understanding of these costs is important for understanding how differences in animal mating systems, and the evolutionary forces that shape them, are related to whole-organism physiology.

Efforts to understand the considerable heterogeneity in gamete production within and between the sexes has generally taken place in the context of life history theory. Among males, differences among species in the size of gonads and/or sperm, and rates of sperm production, are typically attributed to the intensity of postcopulatory sexual selection in the form of sperm competition: Males experiencing greater sperm competition are expected to invest relatively more biomass in gonads and produce sperm at a relatively higher rate [3], [7], [8], [9], [10]. In females, no similar theory has been proposed to explain differences in the energy expended for gonad biomass and gamete production, but the expectation from life history theory is that females produce the optimal size and number of gametes at a rate that maximizes lifetime reproductive success [2], [3], [11]. Between the sexes, the amount of energy invested in gametes by males and females is often assumed to differ, since females aim to maximize offspring survival, whereas males aim to inseminate as many females as possible [2], [3]. However, little consideration has been given to energetic limitations on the production of gamete biomass that may be imposed through constraints on whole-organism metabolism. Moreover, broad-scale interspecific comparisons of the energetic investment in gametes are rare for females and almost non-existent for males (but see [9], [12]).

Here we present a broad-scale comparative study that quantifies two key features of energetic investment in gametes by males and females for diverse species (i.e. invertebrates, reptiles, amphibians, fishes, birds, and mammals) that vary tremendously in their life histories. First, we compare the biomass allocation to gonads in males and females across a broad range of body sizes and assess differences in allocation among taxonomic groups. Like other organs, we expect gonad mass to scale approximately linearly with body mass [13], [14] and for variation about the relationship to be explained by differences in sperm competition among males and differences in clutch size among females. Second, we compare rates of production of gamete biomass in males and females across a broad range of body masses. We hypothesize that, like other rates of biomass production (e.g. growth rate, [15], [16]), the production of gamete biomass should occur at a rate proportional to whole-organism metabolic rate. This presumes that the production of gamete biomass is a function of both gonad mass and gonad metabolic rate and that gonad metabolic rate is proportional to whole-organism metabolic rate. Thus, we expect gamete biomass production rates to scale as a power law with body mass with an exponent of about ¾, as is often observed for whole-organism metabolic rate [16], [17], [18], [19], but see [20], [21], [22]. Since males must produce seminal fluid in addition to sperm, we also assess male investment in ejaculate biomass production (i.e. gametes + seminal fluid). We then quantify the amount of energy devoted to egg, sperm, and ejaculate biomass production relative to basal metabolic rate. Finally, we consider whether residual sperm or egg biomass production rates are related to residual gonad mass. In the case of sperm, a positive relationship between residual sperm biomass production rates and residual testes mass would be consistent with sperm competition theory.

## Results and Discussion

### Investment in Gonads

**Figure 1:**
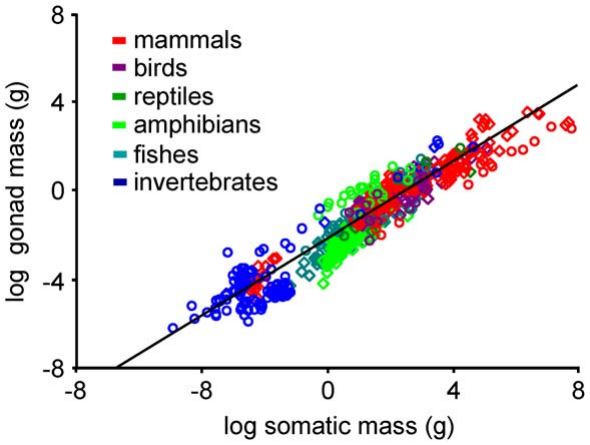
Relationships between gonad and soma mass. The logarithm of gonad mass (g) versus the logarithm of soma mass (g) for males (diamonds) and females (circles).

Across all species, gonad mass (M_g_) scaled somewhat less than linearly with soma mass (M_i_) for both males and females (Fig. 1; logM_g_ = −2.16+0.89 logM_i_, 95%CI_b_: 0.86–0.91, r^2^ = 0.89, p<0.0001), with no significant difference in either the slope or intercept between the sexes (homogeneity of slopes ANCOVA: F_3,639_ = 1768.50; F_Mi_ = 4670.07, F_sex_ = 0.04, F_Mi*sex_ = 0.64). Thus, body mass alone explained 89% of the variation in gonad mass across all taxonomic groups. Including taxonomic group as a covariate increased the amount of variation explained to 91% and revealed statistically significant differences in slopes and intercepts among groups (Table 1). However, in most groups, the slope of the gonad mass-body mass relationship did not differ significantly from 1, though amphibians showed a slightly steeper relationship (b = 1.27, 95%CI_b_: 1.08–1.44) and birds showed a slightly shallower relationship (b = 0.71, 95%CI_b_: 0.59–0.83). Thus, the scaling of gonad mass with body mass was similar across groups and between the sexes, indicating that males and females of diverse species invest similarly in reproductive tissue biomass. This scaling is quite similar to scaling relationships previously observed for other organs, with the possible exception of the brain [13], [14]. This suggests that any previously hypothesized effect of sperm competition on allocation to gonad mass, which would be reflected in the variance about the gonad-soma relationship, is of secondary importance relative to the constraints imposed by whole-organism energetics [4], [6], [7], [8].

**Table 1:** Gonad mass vs. somatic mass by taxon.

effect	n	intercept (95%CL)	slope (95%CL)
intercept		**−2.12 (−2.30 – −1.94)**	
mammals	168	**0.41 (0.18 – −0.63)**	**0.76 (0.82 – 1.06)**
birds	97	**0.50 (0.18 – 0.83)**	**0.71 (0.59 – 0.83)**
reptiles	3	−0.21 (−1.02 **–** −0.60)	**0.96 (0.0.66–1.25)**
amphibians	100	**−0.52 (−0.77 – −0.27)**	**1.27 (1.08 – 1.44)**
fishes	98	**−0.25 (−0.48 – −0.02)**	**0.94 (0.82 – 1.06)**
invertebrates	16	**(zeroed)**	**0.93 (0.86 – 1.00)**

Separate slopes ANCOVA statistics for the relationship between the logarithm of gonad (g) and soma mass (g) by taxon. Significant effects in bold; whole model: r^2^ = 0.91, p<0.0001, F_11,631_ = 559.82; F_taxon_ = 17.78, F_Mi_ = 156.97.

### Gamete Biomass Production Rates

**Figure 2:**
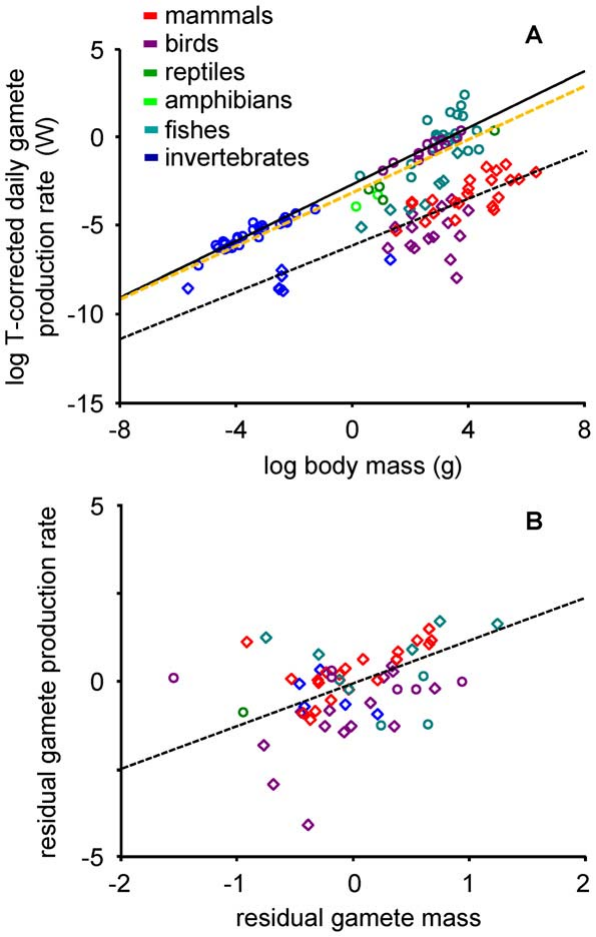
Relationships between gamete biomass production rates, body size, and gonad mass. (**A**) The logarithm of temperature-corrected daily sperm biomass production rates (W; diamonds, dashed black line) and the logarithm of daily egg biomass production rate (W; circles, solid line) versus the logarithm of body mass (g). The relationship between metabolism and body mass for ectotherms at 20°C [13] is plotted for comparative purposes (dashed orange line). (**B**) Residual daily gamete biomass production rates (from a log-log plot of daily gamete biomass production rates versus body mass) versus residual gonad mass (from a log-log plot of gonad mass versus body mass) males (diamonds, dashed line) and females (circles).

Across all species, rates of gamete biomass production in males and females scaled similarly to each other and sub-linearly with body mass (Fig. 2A), indicating that mass-specific rates of gamete biomass production are greater in smaller-bodied species. Specifically, sperm biomass production rates (W) scaled with body mass raised to the 0.66 power and egg biomass production rates (W) scaled to the 0.80 power (separate slopes ANCOVA: F _3,119_ = 277.35, r^2^ = 0.87, p<0.0001; log(sperm biomass production)  = −6.09+0.66 log(body mass (g)) (95%CI_b_: 0.55–0.77); log(egg biomass production)  = −2.66+0.80 log(body mass (g)) (95%CI_b_: 0.73–0.87)). As hypothesized, the slopes of these relationships did not differ significantly from the ¾-power scaling of metabolic rate with body mass. Thus, like other rates of biomass production, a roughly constant fraction of a species' metabolism is devoted to the production of gamete biomass. Notably, these results indicate that, on a mass-specific basis, the biomass allocated to sperm or egg production per lifetime is approximately invariant. In other words, since mass-specific gamete biomass production scaled approximately as M^−¼^ and lifespan scales approximately as M^¼^, lifetime gamete biomass production scales as M^0^. Thus, on average, species invest about the same fraction of their lifetime energy budget in the production of gamete biomass, regardless of the specific life-history strategy they might employ to enhance fitness.

In terms of the energetic cost of gamete biomass production, differences between males and females were substantial. Specifically, our analysis indicates that males and females devoted about 0.1 and 300% of the energy used for basal metabolism to the production of gamete biomass respectively. Thus, the cost of egg production was roughly 3.5 orders of magnitude higher than the cost of sperm production. This difference could not be attributed to males' investment in ejaculate production. Total ejaculate biomass production (W) scaled with body mass as log(ejaculate biomass production)  = −5.52+0.75 log(body mass) (95%CI_b_: 0.60–0.90, r^2^ = 0.75, p<0.0001), such that the cost of ejaculate production (i.e. gametes + seminal fluid) constituted only 0.4% of basal metabolism. Thus, while the cost of ejaculate production was approximately 4-fold higher than that of sperm production alone, egg production rates remained nearly 3 orders of magnitude higher than ejaculate production rates. Still, like rates of gamete biomass production, ejaculate biomass production rates scaled to the ¾-power of body mass and were therefore proportional to whole-organism metabolism.

For both males and females, our estimates of the energetic cost of gamete biomass production across species are consistent with the limited amount of previous work on individual species or species groups. For males, our estimate of the cost of ejaculate production (0.4% of BMR) is similar to that previously reported for Japanese macaques (0.8–6%; [23]). For females, our estimate of the energetic cost of egg production (∼300% of BMR) is in close agreement with the range of estimates (∼20% to ∼200%) that have been presented for various species of birds [24], [25], [26]. If we assume that eggs are produced throughout the year, rather than only during the breeding season, our estimate of the energetic cost of egg biomass production decreases to about 50% of BMR. Our findings for females also compliment those of a recent study that found that mass-specific reproductive biomass production rates scaled as body mass to the power of −0.37 in mammals, when reproductive biomass was measured in terms of the mass of newly weaned young [27].

While body mass explained much of the variation in rates of gamete biomass production in both sexes (63 and 94% in males and females, respectively), residual testes mass (from a log-log regression of testes mass on body mass) explained a small amount of the residual variation in gamete biomass production rates in males (log(residual sperm production)  = −0.09+1.23 log(residual testes mass (g)), (95%CI_b_: 0.56–1.89, r^2^ = 0.24, p<0.001; Fig. 2B). These results might be viewed as consistent with predictions from sperm competition theory, though the relationship was weak and highly variable among groups. Based on limited data, a separate slopes ANCOVA indicated that birds and mammals had statistically significant, positive relationships, whereas fishes and invertebrates did not show statistically significant relationships (F_7,37_ = 11.78, r^2^ = 0.63, p<0.0001; birds: b = 1.98 (95%CI: 1.06–2.89), p<0.001, n = 14; mammals: b = 1.02 (0.28–1.75 95%CI), p<0.0001, n = 21; fishes: n = 5; invertebrates: n = 5). Note, however, that residual egg biomass production rates were not related to residual ovary mass (Fig. 2B).

### Conclusions

Our results provide insights regarding the investment by males and females in gonad and gamete biomass. Both within and between the sexes, investment in gonad biomass was quite similar across species. This is reflected in the similarity in both the slopes and intercepts of the scaling relationships of testes and ovary mass with body mass. With respect to gamete biomass production, the story appears to be quite different. Within each sex, the production of gamete biomass scaled sub-linearly with body mass across species in about the same way as whole-organism metabolic rate. However, between the sexes, rates of gamete biomass production were two to four orders of magnitude higher in females. This presents an interesting question for future research as it suggests that mass-specific rates of gamete biomass production, and perhaps mass-specific rates of metabolism in general, were much higher in ovaries than testes.

More generally, the results presented here raise questions regarding hypotheses aimed at explaining differences in reproductive investment between males and females based on sexual selection. For example, sperm competition theory and models of parental investment generally assume that gamete biomass production is proportional to gonad mass [8]. However, our results indicate that rates of production of gamete biomass are roughly proportional to whole-organism metabolic rate and that gonad mass is of secondary importance. As such, these results point to the need to integrate theory on sexual selection and life history with fundamental principles of organism-level physiology when addressing questions related to costs of sexual reproduction. Quantifying the energetic cost of gamete production and relating it to individual metabolic rate, as we have done here, may ultimately be helpful in quantifying tradeoffs in parental investment given the finite energy budget of individuals.

## Materials and Methods

Gonad and somatic tissue mass data were obtained from the literature for 656 species (Table S1). Estimates of daily rates of egg (72 species) and sperm (51 species) biomass production during the breeding season (g/d) were either obtained directly from the literature, or calculated by multiplying estimates of gamete mass by published estimates of the number of gametes produced per day (Table S2). When production rates were presented as annual rates of production, they were converted into daily rates of production by dividing by breeding season length (d/yr), as obtained from the literature (see Table S2). Gamete mass was estimated from gamete volume by assuming biomass density was equivalent to that of water (1 g/mL; [13]). When not reported directly (in many cases), total sperm volume was estimated using the volume of each of the three primary sperm features (i.e. head, midpiece, and tail; J. F. Gillooly, H. B. Vander Zanden, & A. Hayward, unpublished data). The volume of a given sperm feature was determined using linear dimensions and the approximate shape of each feature as reported or inferred from the literature. When shape was not reported, the equation for the volume of an ellipsoid or spheroid was used to calculate head and midpiece volume (depending on the number of dimensions available) and the equation for a cylinder was used to calculate tail volume. When insufficient data were available to calculate sperm volume for a given species, volume was either approximated using sperm volume(s) from closely related taxa or estimated using observed allometric relationships between total volume and head volume for mammals (log total volume  = 0.56+0.74 * log head volume, r^2^ = 0.80, p<0.0001) or total volume and midpiece volume for birds (log total sperm volume  = 1.07+0.23 * midpiece volume, r^2^ = 0.45, p<0.05) (see Table S2). Daily ejaculate production rates were estimated from previously published data using ejaculate volume and the frequency of ejaculation or using the concentration of sperm per ejaculate and the number sperm produced per day (see Table S2).

Gamete mass expressed in grams of carbon was converted to dry weight (g) using a conversion factor of 0.4. Dry weight was converted to wet weight (g) using a conversion factor of 0.25 g dry weight/g wet weight [13]. Biomass production data were converted from g/d to J/d using a conversion factor of 7.0×10^6^ J/g and then into Watts by dividing by 86400 s/d in order to compare the amount of energy devoted to gamete biomass production with basal metabolic rate of ectotherms at 20°C (metabolic rate (W) = 0.14 • mass (kg)^0.751^ [13]. Gamete biomass production was assumed to have occurred at ambient environmental temperature for ectotherms and at 38°C and 40°C for mammals and birds, respectively. Rates of production were corrected to 20°C by assuming production increased exponentially with temperature using the Boltzmann-Arrhenius factor (*e* ^−*E/kT*^), where *E* is the average activation energy of the respiratory complex (∼1.04×10^−19^ J (0.65 eV)), *k* is Boltzmann's constant (1.381×10^−23^ J•K^−1^ (8.62×10^−5^ eV•K^−1^)), and *T* is absolute temperature in Kelvin [28]. This is equivalent to assuming a Q_10_ of about 2.5.

Statistical analyses were carried out in Statistica 8.0. Homogeneity of slopes analysis was used to determine whether the relationship between gonad and soma mass differed among sexes or groups. Separate slopes ANCOVA was used to compare the scaling of gonad mass with soma mass among groups, and to evaluate differences in the scaling of gamete production rates and body mass between the sexes. Bivariate OLS regressions were used to evaluate the relationship between ejaculate biomass production rate and body mass and between residual gamete biomass production rates and residual testes mass. Differences in the scaling of residual sperm biomass production rates with residual testes mass among groups were evaluated using a separate slopes ANCOVA.

## Acknowledgments

We wish to thank Jennifer Parker and Hannah B. Vander Zanden for their efforts in compiling and collating portions of the data presented here.

## Supporting Information

Table S1 **Gonad and soma mass data.** Raw data and sources for gonad and soma mass data used in this study.
(XLSX)

Table S2 **Gamete biomass production rate data.** Raw data and sources for egg and sperm biomass production rate data used in this study.
(XLSX)
